# Beneficial Impact of Semicarbazide-Sensitive Amine Oxidase Inhibition on the Potential Cytotoxicity of Creatine Supplementation in Type 2 Diabetes Mellitus

**DOI:** 10.3390/molecules25092029

**Published:** 2020-04-27

**Authors:** Dimitri Papukashvili, Nino Rcheulishvili, Yulin Deng

**Affiliations:** 1 School of Life Science, Beijing Institute of Technology, Beijing 100081, China; dimitri@bit.edu.cn (D.P.); nino@bit.edu.cn (N.R.); 2 Beijing Key Laboratory for Separation and Analysis in Biomedicine and Pharmaceuticals, Beijing 100081, China

**Keywords:** creatine, T2DM, SSAO, caffeine, ZAG/Zn, Cu, Fe, histamine/histidine, vitamin D

## Abstract

Creatine supplementation of the population with type 2 diabetes mellitus (T2DM) combined with an exercise program is known to be a possible therapy adjuvant with hypoglycemic effects. However, excessive administration of creatine leads to the production of methylamine which is deaminated by the enzyme semicarbazide-sensitive amine oxidase (SSAO) and as a result, cytotoxic compounds are produced. SSAO activity and reaction products are increased in the serum of T2DM patients. Creatine supplementation by diabetics will further augment the activity of SSAO. The current review aims to find a feasible way to ameliorate T2DM for patients who exercise and desire to consume creatine. Several natural agents present in food which are involved in the regulation of SSAO activity directly or indirectly are reviewed. Particularly, zinc-α2-glycoprotein (ZAG), zinc (Zn), copper (Cu), histamine/histidine, caffeine, iron (Fe), and vitamin D are discussed. Inhibiting SSAO activity by natural agents might reduce the potential adverse effects of creatine metabolism in population of T2DM.

## 1. Introduction

**Figure 1:**
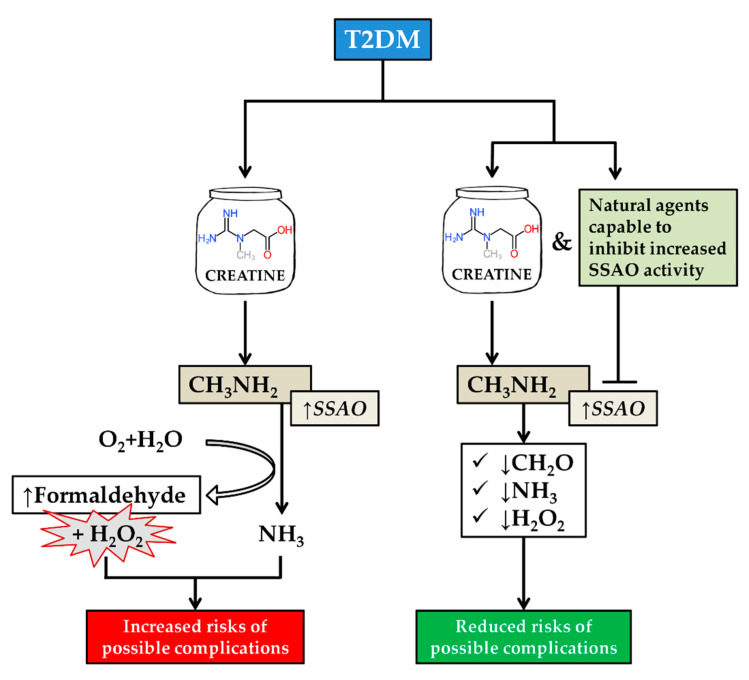
An illustration of the role of natural agents on SSAO activity throughout the supplementation of creatine in T2DM patients. Upregulation of SSAO activity and creatine related methylamine production which is a substrate of SSAO elevates formaldehyde, ammonia, and H_2_O_2_ in T2DM patients which causes possible complications; Additional ingestion of natural agents capable to inhibit increased SSAO activity diminishes the production of formaldehyde, ammonia, and H_2_O_2_ and risks of possible complications are reduced. Notes: T2DM, type 2 diabetes mellitus; SSAO, semicarbazide-sensitive amine oxidase; ↑, upregulation; ↓, downregulation; CH_3_NH_2_, methylamine; CH_2_O, formaldehyde; H_2_O_2_, hydrogen peroxide; NH_3_, ammonia.

Diabetes is a chronic hyperglycemic disease. The population with type 2 diabetes mellitus (T2DM) is going to reach an alarming level soon [1,2]. It is most prevalent in subjects older than 45 years [3,4], however, the number of diabetes cases has increased in young people too [5]. To improve the glycemic condition, which is the main problem in diabetes condition, physical exercise therapy is necessary for all ages [6,7]. Creatine is a naturally produced nitrogenous molecule that improves physical strength in vertebrates [8]. Creatine facilitates recycling of adenosine triphosphate (ATP)- the energy currency of the cell, therefore, represents the key component of energy metabolism in muscle [9]. In the body, approximately 1 g/day of creatine is synthesized in the liver, pancreas, and kidneys from arginine, glycine, and methionine [10,11]. It is also obtained by ingesting foods such as red meat and seafood [12]. The body converts creatine into phosphocreatine and stores it in the muscles where it can be used as an energy source [13]. Furthermore, creatine is also made synthetically [14] and is one of the most popular natural supplements [15,16,17,18] used for improving muscle power, strength, and gaining lean mass [17,18,19,20]. For that reason, creatine and physical exercise are often referred together. Excessive creatine supplementation can elevate methylamine levels [21,22,23,24,25] which, as a substrate of semicarbazide-sensitive amine oxidase (SSAO), is oxidatively deaminated [26,27] and cytotoxic formaldehyde is produced [28,29]. Nevertheless, creatine supplementation does not impair kidney function in healthy individuals [30] and even in animals with pre-existing renal failure [31]. Gualano et al. showed that creatine supplementation combined with exercise alleviated glycemic conditions in T2DM [13]. Although creatine intake has a hypoglycemic effect, on the other hand, it might promote certain health risks accompanying its supplementation. SSAO-mediated deamination products, such as aldehydes, hydrogen peroxide (H_2_O_2_), ammonia, are produced, therefore, unfavorable health consequences might arise. Notably, SSAO activity in the blood plasma of diabetic patients is upregulated [32,33,34,35,36]. SSAO is a copper (Cu)-containing enzyme. There is evidence that serum Cu levels are elevated in T2DM cases [37]. Based on the aforementioned information, we assume that the prevention of possible adverse outcomes of creatine supplementation in T2DM patients is needed. SSAO inhibition may have a beneficial impact on creatine supplementation in diabetic patients. There are numbers of already well-known inhibitors capable to inhibit SSAO activity but most of them are toxic to some extent [38]. Interestingly, there are some natural compounds capable to lower increased SSAO activity. In this review, natural, relatively accessible, and possibly advantageous agents with respect to SSAO inhibition are discussed. The role of inhibitive effects on SSAO throughout the supplementation of creatine in T2DM patients is illustrated in Figure 1.

## 2. Creatine Supplementation and Type 2 Diabetes Mellitus

Creatine is an essential compound with a positive influence on brain function [39,40,41] and muscle performance [42]. Its estimated daily intake without a special diet is approximately 1 g/day, while 25–30 g/day can be achieved via special supplementation including a high protein diet [14]. It is accumulated in the body approximately 120–140 g in 70 kg young males, although, this amount varies individually according to the muscle fiber types, fat-free mass, etc. [15]. Because of ergogenic aids, it represents a popular nutritional supplement for athletes [10,15,17,43]. Creatine monohydrate and nitrate are prominent supplements used for improving exercise performance [44]. Other less popular supplements, as well as dietary sources, are presented in Table 1. Most of the studies are executed on creatine monohydrate. The effective loading dose of creatine monohydrate is 0.3 g/kg daily for 5–7 followed by the maintenance, 0.03 g/kg daily for approximately 6 weeks [45]. After administration, it is transported into the cells by the creatine transporter 1 (CreaT1). Afterward, it is being degraded spontaneously and a waste product creatinine is produced while via the action of creatinase sarcosine is generated; by sarcosine reductase methylamine is produced and then SSAO-mediated oxidation takes place [22].

Due to oral supplementation, creatine levels in the body are increased which is followed by the saturation of creatine into various cells or by clearing it from the blood via renal filtration [11]. Adverse effects may appear as a result of creatine supplementation overdosing by patients with potential risks or pre-existing disorders of renal diseases [23,55]. Methylamine, the metabolic product of creatine, is regarded to play an essential role in SSAO activity and, as a result, cytotoxic compounds are produced [56,57]. Nevertheless, methylamine produced by creatine supplementation (up to 20 g/day) in healthy subjects, is within normal limit values [11], meaning that, methylamine produced via intake of creatine up to 20 g/day does not impair kidney function [23]. Interestingly, creatinine is a break-down product of creatine and low serum creatinine is associated with the increased risk of T2DM [58,59]. Indeed, Nie et al. found creatine levels to be considerably decreased in T2DM rats [60]. Moreover, creatine consumption in T2DM cases along with an exercise program is known to be beneficial for blood glucose level regulation and glucose transporter protein, GLUT-4 expression [61,62]. Gualano et al. demonstrated that creatine supplementation has no adverse effect on kidney function in individuals with T2DM [61]. Instead, it even improves insulin sensitivity in animal models [62] and T2DM patients [63,64]. Therefore, creatine may be proposed as a beneficial sports supplement for T2DM. However, it might have negative effects concerning SSAO activity alteration.

## 3. Why Is Semicarbazide-Sensitive Amine Oxidase Activity Elevated in Type 2 Diabetes Mellitus?

**Figure 2:**
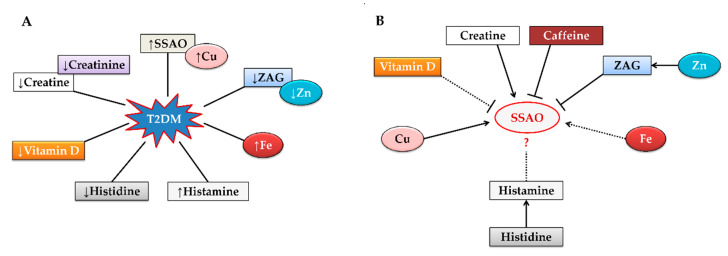
(**A**) The concurrence of certain natural agents in plasma of T2DM patients. (**B**) The impact of certain natural agents on SSAO activity. Notes: T2DM, type 2 diabetes mellitus; SSAO, semicarbazide-sensitive amine oxidase; Cu, copper; ZAG, zinc-α2-glycoprotein; Zn, zinc; Fe, iron; ↑, upregulation; ↓, downregulation; ――, direct or indirect involvement in SSAO activity; ……, possible involvement in SSAO activity.

T2DM is a pathology that implies impaired glucose transport in the cells [3]. The compensatory mechanisms are involved in this process. SSAO is overexpressed and its activity is increased [32]. Accordingly, the formation of cytotoxic compounds- formaldehyde, ammonia, and H_2_O_2_, which may have a deleterious influence on the body, is elevated [21,22]. H_2_O_2_ is involved in insulin mimicry that enhances glucose transport [65]. The mentioned mechanism works to compensate for the worsen glycemic condition which is common for diabetes. However, compounds with adverse effect are formed simultaneously that is associated with diabetes complications- cardiovascular diseases, stroke, etc. [66]. Thus, the scientists assumed that inhibiting SSAO activity will diminish the risks and complications in diabetes [38]. Certain natural compounds and elements discussed in this article and their impact on SSAO activity are given in Figure 2.

## 4. Role of Semicarbazide-Sensitive Amine Oxidase in Creatine Metabolism

SSAO is expressed in plasma, on the surface of endothelial cells and adipocytes [67]. Its substrate, methylamine, is not as toxic as it becomes in the presence of SSAO that induces deamination. Subsequently, cytotoxic compounds are produced [21,68]. The accumulation of those compounds in mice was found as a result of high concentrations of methylamine and enhanced activity of SSAO [21,69]. Poortmans et al. studied 21 g/day of creatine monohydrate oral supplementation in 20 healthy male individuals and revealed that high dose loading of creatine in a short period of time increased excreted levels of methylamine and formaldehyde, 9.2-fold and 4.5-fold in the urine, respectively. However, this level of methylamine is still below the upper limit value of the normal range [22]. Therefore, creatine administration within the recommended doses by healthy subjects does not pose a risk. Besides, Gualano et al. reported a positive effect of creatine supplementation and aerobic training on glucose tolerance in sedentary healthy males [70].

## 5. Semicarbazide-Sensitive Amine Oxidase Mediated Creatine Impact on Type 2 Diabetes Mellitus

Diabetes mellitus is a metabolic disease when blood glucose levels rise from the normal range due to hormone insulin-related disorders, such as obesity [2]. Obesity is an unhealthy condition that includes excess fat accumulation in the body. This metabolic disorder along with an inactive lifestyle and an unhealthy diet increases T2DM cases over the last decade [71]. Notably, according to the abovementioned, the enzyme SSAO levels are increased in this medical condition [32]. Thus, consuming creatine in diabetes will increase the production of methylamine [24] which will be oxidized by SSAO and converted into cytotoxic compounds [21,55,72]. These substances appear to be harmful to the body in case of excessive accumulation. Interestingly, formaldehyde is found to induce cognitive impairments in diabetes [73]. It is known that the concentration of methylamine is upregulated in some physiological and pathological conditions—the former implies pregnancy and the latter—diabetes mellitus, among many others [74]. SSAO activity in both type 1 and type 2 diabetic patients may be upregulated due to augmentation of its substrates concentrations [75]. It is also noteworthy that in diabetic patients with cardiovascular complications, elevated plasma activity of SSAO is reported [21,76].

## 6. Promising Agents Involved in Regulating Semicarbazide-Sensitive Amine Oxidase Activity

### 6.1. Ability of Caffeine to Inhibit Semicarbazide-Sensitive Amine Oxidase and Its Simultaneous Administration with Creatine

In consonance with all the information mentioned above, it can be presumed that an effective solution is needed for the prevention of adverse outcomes in diabetic patients. Hence, SSAO inhibition aids patients with diabetes to decrease the levels of toxic compounds. Caffeine is a natural substance with antioxidant capacity commonly consumed worldwide in daily life [77,78]. It has the ability of inhibiting SSAO as it contains an imidazole ring [68,79]. Most of the inhibitors of this enzyme react on the imidazoline-binding inhibitory site of SSAO [79]. Moreover, caffeine was found to be ingested by T2DM patients more than by non-diabetics to decrease disease-associated drowsiness [80,81]. Studies evidenced the positive impact of caffeine intake in the population with T2DM [82,83]. Neves et al. demonstrated that consumption of caffeine from coffee had a protective effect on mortality of women with diabetes [84]. Additionally, Wistar and Goto-Kakizaki (GK) rats were administered with 1 g/L of caffeine dissolved in drinking water for 4 months. A favorable influence on the spatial memory which is associated with hippocampus normal function was observed in T2DM animals [85]. Apart from that, there are numbers of human and animal studies regarding the beneficial properties of caffeine and caffeinated products for promoting weight loss [86,87]. Da Silva et al. have studied the influence of 1.5 mg/kg caffeine intake on blood glucose levels in T2DM individuals throughout the exercise. This dose showed to be efficient for glucose-intolerance management [88]. Olivieri and Tipton have shown that 0.1–10 mM caffeine can inhibit SSAO activity IC_50_ = 0.8 ± 0.3 mM in bovine serum [79]. This is a quite wide range, thus, more human studies are necessary to understand the safety and the most effective amount of caffeine to inhibit the enzyme significantly. Moreover, Che et al. also reported its inhibitory effect (50 mg/kg/day, i.p.) influence on SSAO activity in various tissues of Wistar rats after administration for 10 and 25 days [68]. Caffeine is relatively safe in most of the commercially available foods and drinks [89,90].

Inhibition of SSAO activity can protect cells from the cytotoxic damage associated with SSAO-methylamine reaction products in T2DM patients. Thus, caffeine consumption might balance the activity of SSAO in people with T2DM who supplement creatine. However, future researches are needed with a larger number of individuals as there are articles indicating caffeine unprepossessing influence on blood glucose levels in type 1 and type 2 diabetes mellitus [91,92,93]. This can be explained by caffeine’s inhibitive ability on SSAO activity. Consequently, less H_2_O_2_ is produced and, therefore, the level of insulin mimicry decreases which worsens glucose tolerance.

In diabetes creatine concentrations are significantly low [61]. Creatine supplementation along with exercise training improves the glycemic condition in T2DM as well as balances creatine levels [94]. SSAO-mediated cytotoxicity side effects exist, therefore, the intake of caffeine in this circumstance is logical. The safe daily dose of caffeine is 400 mg per day which corresponds to the amount presented in approximately up to 4 cups of coffee [95]. There is evidence of creatine and caffeine benefits when ingested combined in healthy humans [96]. Twelve healthy male subjects were ingested by creatine with 5 days abstinence from caffeine and then were given caffeine. The study revealed that caffeine had a beneficial impact on sprint performances when consumed after loading of creatine [97]. Nonetheless, most of the researches refer to their non-ergogenic effect [98,99,100,101,102]. As there are no articles published with respect to creatine and caffeine simultaneous supplementation neither by humans nor animal models with diabetes, it remains unclear. Moreover, caffeine consumption diminishes glucose uptake [103,104] as it reduces SSAO activity, and, as a result, the glycemic condition is exacerbated. The impact of caffeine and creatine separate consumption on healthy and diabetic conditions is presented in Table 2. Studies including treatment with creatine-caffeine concurrent supplementation in healthy subjects/animals are given in Table 3.

### 6.2. Histamine/Histidine- Good or Bad for Type 2 Diabetes Mellitus?

Histamine is a neurotransmitter in the body that contains an imidazole ring. The activity of this bioamine is closely related to lipolysis and SSAO metabolism. It improves the condition of leptin-resistance, thus plays an important role as an anorexigenic agent [112]. Although the enzyme diamine oxidase (DAO) is responsible for oxidizing histamine, SSAO activity is also found to be related to the catalysis of histamine oxidation [113]. Importantly, histidine, a precursor amino acid for histamine was observed to improve insulin sensitivity and ameliorate metabolic syndrome, hence, by regulating hepatic glucose output, it can assist in the glycemic control in diabetes [114]. Kimura et al. demonstrated that histidine can augment the suppression of glucose production in T2DM [115]. As the dietary histidine is suggested to ameliorate glycemic condition via its favorable effects on insulin sensitivity, administration of histidine-rich nutriments, which are mostly protein-containing food, can be considered [114]. Moreover, histidine levels along with creatine concentration are found to be significantly diminished in rats with T2DM [61]. Based on the evidence, Coppari et al. hypothesized the therapeutic effects of leptin treatment- a hormone which diminishes fat storage in adipocytes, for diabetes [116]. Interestingly histamine is known to ameliorate leptin-resistance [112] which occurs in diabetes [116]. However, increased plasma histamine levels are observed in diabetic patients [117]. Hence, the data about the histamine/histidine impact on T2DM remain conflicting [118]. Apart from the involvement in SSAO activity regulation, histamine/histidine concern remains unclear up till now.

### 6.3. Zinc-α2-glycoprotein/Zinc

Along with the referred substances, zinc-α2-glycoprotein (ZAG) —a major plasma protein is found to owe SSAO inhibitive capacity via binding the enzyme at the non-catalytic site and reducing its activity non-competitively [119]. In the body, it is a responsible factor in stimulating lipolysis [120]. Gong et al. studied human subjects as well as mice and demonstrated that the concentration of serum ZAG was inversely proportional to the body fat in humans and mice [121]. Changes in ZAG levels are closely related to obesity [122] and the associated comorbidities such as diabetes, obesity, polycystic ovary syndrome [123,124], non-alcoholic fatty acid liver disease (NAFLD), etc. [125]. Particularly, ZAG exhibits the attenuation of diseases associated with obesity, e.g., NAFLD [125,126]. NAFLD is a prevalent complication of T2DM [127]. ZAG can play a certain role in T2DM. Indeed, studies have demonstrated that this glycoprotein is connected with hyperglycemia, insulin resistance, and other health conditions common in diabetes [128]. Circulating ZAG in T2DM patients is presented in lower concentrations than in control subjects [129]. In addition, the elevated expression of ZAG is related to weight gain attenuation [130] which is a common condition for subjects with T2DM [131]. Furthermore, ZAG levels in obese subjects are low as well [132].

In accordance with all the mentioned information, it would be prudent to find a strategy of increasing ZAG levels in the body. Interestingly, some studies demonstrated that the concentration of zinc (Zn), which is a constituent of ZAG, was found to be decreased in the serum of diabetic patients [37,133,134]. Moreover, Zn is evidenced to have a beneficial impact on diabetes [135,136]. The studies give the rationale that Zn dietary intake might reduce T2DM risk and ameliorate this health complication. Agents positively affecting the elevation of ZAG respectively have an impact on SSAO through the indirect way, meaning that, increasing ZAG levels are related to diminishing SSAO activity. Thus, moderately increasing the administration of Zn-rich food can be used as a therapeutic strategy as Zn has promising potential in diabetes and, moreover, it might indirectly reduce SSAO activity which is associated with creatine consumption. Creatine and Zn combination as a supplement might merit future development after the necessary number of studies.

### 6.4. Copper

SSAO belongs to Cu-containing primary amine oxidases. The concentrations of Cu and the enzyme itself are found to be positively correlated and are elevated in plasma and serum of diabetic patients [37,137]. Indeed, Yang et al. demonstrated that the expression of Cu-dependent enzymes and SSAO were increased along with the adipocyte differentiation [138]. Diabetes is associated with obesity which, on the other hand, implies excessive number and size of differentiated adipocytes. Moreover, the study conducted on mice showed that the serum Cu status was significantly higher in diabetic than in non-diabetic mice [139]. Squitti et al. demonstrated significantly elevated Cu levels in patients with T2DM as compared to the healthy subjects [140]. Tanaka et al. demonstrated that the Cu-chelating agent tetrathiomolybdate reduced insulin resistance and improved glucose tolerance in diabetic mice [139]. As Cu-chelating agents are not presented in food, avoiding intake of Cu-rich dietary sources may contribute to ameliorating T2DM condition while creatine supplementation.

### 6.5. Role of Dietary Iron Along with Creatine Supplementation in Type 2 Diabetes Mellitus

Iron (Fe) body levels are positively associated with the risk of T2DM [141]. Indeed, the serum concentration was found to be markedly increased in T2DM patients [37]. Dutra et al. investigated the oxidation of aminoacetone along with Fe presence. Aminoacetone represents one of the substrates of SSAO, and its levels are found to be increased in diabetes [142]. With the simultaneous existence of Fe ions, aminoacetone oxidation and H_2_O_2_ formation were enhanced significantly [142]. This suggests that the high Fe intake increases the risk of T2DM [143]. In T2DM H_2_O_2_ production is already augmented via SSAO activity, thus, elevated Fe consumption causes additional cytotoxicity. As in diabetic subjects, Fe levels are furthermore elevated, accordingly, moderate limitation of Fe intake along with creatine supplementation seems to be coherent. Moreover, increased Fe levels are linked to cardiovascular disease which represents diabetes complication [144]. However, Fe deficiency is found in obesity- concomitant disorder of diabetes [145,146]. Apparently, studies in this direction merit the attention to shed light on the impact of simultaneous consumption of Fe and creatine.

### 6.6. Vitamin D

Vitamin D is known to aid the body to ameliorate insulin sensitivity and thus, a glycemic condition in T2DM [147]. Data show that vitamin D deficiency is a basis of diabetes [148]. Low vitamin D levels are found to be associated with many disorders, including T2DM and cardiovascular diseases [149]. The progression of these health disorders involves SSAO activity. Based on the evidence, vitamin D improves vascular function and decreases another amine oxidase—monoamine oxidase-A (MAO-A) levels [150]. Some of MAO inhibitors manifested to inhibit SSAO activity as well [151,152,153] which develops the basis that vitamin D may also influence SSAO activity. Besides, creatine and vitamin D are present together in seafood and dairy products. Moreover, vitamin D along with calcium intake shows an improvement of important antioxidant enzymes in oxidative stress- superoxide dismutase (SOD) and catalase (CAT) activities among other benefits in diabetic rats [154,155]. SOD neutralizes superoxide radicals while CAT is responsible for decomposition of H_2_O_2_ into water and oxygen. The deficiency of these enzymes causes the risk of diabetes as oxidative stress takes place [156,157,158]. It is also observed that vitamin D supplementation increases Zn uptake with possible formation of Zn-vitamin D complex [159]. Although there is no evidence regarding vitamin D and SSAO direct linkage, it can be assumed that creatine supplementation with vitamin D combination in T2DM seems quite promising.

Major dietary sources of vitamin D with the other discussed agents involved in SSAO activity regulation are presented in Table 4. However, the thermal processing of food will decrease the content [160,161], therefore, concurrent supplementation might be considered for a better outcome.

## 7. Conclusions and Future Perspectives

Evincing all the above information, the current article provides a strong basis for studies focusing on the effects of the agents capable to ameliorate SSAO-induced cytotoxicity with respect to creatine in T2DM. Creatine supplementation is related to the modulation of glucose uptake in diabetes, as well as elevation of methylamine deamination products due to increased SSAO activity. Creatine supplementation along with ameliorating SSAO-mediated cytotoxicity with natural agents in dietary sources or supplements appears to be reasonable. The minor toxicity, inexpensiveness, and high consumption in daily life make caffeine easily available strategy to reduce the possible cytotoxic effects of creatine in diabetes.

Nonetheless, creatine and caffeine have contrary effects in terms of hydration- retention, and excretion, respectively. Thus, in case of such combination, intake of a sufficient amount of water should be recommended. Apart from that, Zn as a constituent of ZAG and widely available element in food seems to be promising for diabetes therapy concerning the regulation of ZAG levels and consequently, SSAO inhibition. High dietary Zn intake might be negatively associated with diabetes complications. Besides, studies aiming regulation of Cu concentration in the body need to be considered as a high level of Cu is associated with SSAO increment. Vitamin D might be involved in SSAO activity regulation chain, hence, its supplementation effects along with creatine intake merit further consideration. Limiting dietary Fe administration with creatine supplementation seems rational. Unlike the mentioned agents, histamine and histidine warrant more caution. In obesity histamine and serum SSAO levels are negatively associated, while in diabetes, the opposite result occurs. The molecular mechanism of the histamine and SSAO linkage in diabetes remains unclear. Thus, the investigation of the described agents requires further development. Taken together, counterbalancing the potentially negative impact of creatine in T2DM condition with respect to SSAO by the reviewed agents, may neutralize the cytotoxic effects. Eliminating possible adverse outcomes of creatine supplementation via inhibiting SSAO activity might have a beneficial impact in T2DM population.

## Figures and Tables

**Table 1:** Major dietary sources of creatine.

Creatine Natural and Synthesized Sources	Major Sources	References
Creatine dietary sources	Red meat	Post et al. [46] Kreider et al. [47]
	Dairy products	
	Seafood	
Creatine supplements	Creatine monohydrate	Inácio [48] Buford et al. [49]
	Creatine nitrate	Galvan et al. [44]
	Creatine hydrochloride	Gufford et al. [50]
	Creatine ethyl ester	Gufford et al. [51]
	Buffered creatine	Jagim et al. [52]
	Liquid creatine	Gill et al. [53]
	Creatine magnesium chelate	Selsby et al. [54]

**Table 2:** Impact of caffeine and creatine separate consumption on healthy and diabetic conditions.

Compounds	Healthy Humans/Animal Models									Type 2 Diabetes Mellitus – Humans	
	Weight-Gain	Weight-Loss	(↑) SSAO Upregulation	(↓) SSAO Inhibition	Water-Retention	Increased Urinary-Excretion	Ergogenic Effect	Reducing Insulin Sensitivity	Improving Glucose Tolerance	Anti-Hyperglycemic Effect	Impairing Kidney Function
Creatine	Yes [105] human	No [105] human	Yes [21] animal (healthy)	No [21] animal (healthy)	Yes [105] human	No [105] human	Yes [106] human (healthy)	No [70] human (healthy)	Yes [70] human (healthy)	Yes [64]	No [61]
Caffeine	No [107] human	Yes [107] human	No [68] animal	Yes [68] animal	No [108] human	Yes [108] human	Yes [109] human	Yes [110] animal	No effect on healthy animals Yes (in diabetic animals) [111]	Yes [82] No [86] Yes [105]	No [84]

**Notes:** SSAO, semicarbazide-sensitive amine oxidase; ↑, upregulation; ↓, downregulation.

**Table 3:** Studies including treatment with the combination of Creatine-Caffeine in healthy, non-diabetic subjects/animals.

Title of Study	Study Object	Used Doses	Summary	References
Caffeine is ergogenic after supplementation of oral creatine monohydrate	Humans	Creatine - 0.3 g/kg/day Caffeine - 5 mg/kg/day	Caffeine ingestion has an ergogenic effect in trained males after 6 days of creatine loading administration and caffeine abstinence	Doherty et al. [96]
Effect of caffeine ingestion after creatine supplementation on intermittent high-intensity sprint performance	Humans	Creatine - 0.3 g/kg/day Caffeine - 6 mg/kg/day	Caffeine ingestion after creatine loading for 5 days increased the strength of physically active men as compared to the control group	Lee et al. [97]
Effects of coffee and caffeine anhydrous intake during creatine loading	Humans	Creatine - 5 g 4 times a day Caffeine - 300 mg 4 times a day	These findings suggest that neither creatine alone, nor in combination with caffeine or coffee, significantly affected performance compared to placebo.	Trexler et al. [99]
The effects of a high dosage of creatine and caffeine supplementation on the lean body mass composition of rats submitted to vertical jumping training	Rats	Creatine - 0.430 g/kg/day (loading), 0.143 g/kg (maintenance) Caffeine −15 mg/kg/day	Creatine and caffeine combination did not influence on lean body mass in sedentary and exercised rats while caffeine administration reduced fat	Franco et al. [102]

**Table 4:** Major dietary sources of caffeine, histidine, Zn, and vitamin D. Cu-chelators are not presented naturally in food.

Promising Agents to Combine with Creatine	Major Dietary Sources	References
Caffeine	Coffee	[162]
	Tea	
	Cocoa	
	Chocolates	
Histidine	Meat and meat products	[163]
	Grain products	
	Dairy products	
	Vegetables	
	Seafood	
	Egg	
	Beans	
	Nuts	
Zn	Meat	[164]
	Legumes	
	Poultry	
	Dairy products	
	Nuts	
	Seafood	
Cu	Legumes	[165]
	Mushrooms	
	Chocolate	
	Nuts	
	Beef	
	Seafood	
Fe	Liver	[166]
	Beef, pork, lamb	
	Beans	
	Cereals	
	Seafood	
	Nuts	
	Peas	
Vitamin D	Fish	[167]
	Mushrooms	
	Egg	
	Liver	
	Beef	
	Chicken breast	
	Dairy products	
	Soybeans	
